# Detection of influenza virus and *Streptococcus pneumoniae* in air sampled from co-infected ferrets and analysis of their influence on pathogen stability

**DOI:** 10.1128/msphere.00039-23

**Published:** 2023-05-31

**Authors:** Andrea J. French, Nicole C. Rockey, Valerie Le Sage, Karina Mueller Brown, Meredith J. Shephard, Sheila Frizzell, Mike M. Myerburg, N. Luisa Hiller, Seema S. Lakdawala

**Affiliations:** 1 Department of Microbiology and Molecular Genetics, University of Pittsburgh School of Medicine, Pittsburgh, Pennsylvania, USA; 2 Department of Biological Sciences, Carnegie Mellon University, Pittsburgh, Pennsylvania, USA; 3 Division of Pulmonary, Allergy, and Critical Care Medicine, University of Pittsburgh, Pittsburgh, Pennsylvania, USA; 4 Department of Microbiology and Immunology, Emory University School of Medicine, Atlanta, Georgia, USA; University of Michigan, Ann Arbor, Michigan, USA

**Keywords:** influenza, *Streptococcus pneumoniae*, aerosols, stability, survival, persistence

## Abstract

Secondary infection with *Streptococcus pneumoniae* has contributed significantly to morbidity and mortality during multiple influenza virus pandemics and remains a common threat today. During a concurrent infection, both pathogens can influence the transmission of each other, but the mechanisms behind this are unclear. In this study, condensation air sampling and cyclone bioaerosol sampling were performed using ferrets first infected with the 2009 H1N1 pandemic influenza virus (H1N1pdm09) and secondarily infected with *S. pneumoniae* strain D39 (Spn). We detected viable pathogens and microbial nucleic acid in expelled aerosols from co-infected ferrets, suggesting that these microbes could be present in the same respiratory expulsions. To assess whether microbial communities impact pathogen stability within an expelled droplet, we performed experiments measuring viral and bacterial persistence in 1 µL droplets. We observed that H1N1pdm09 stability was unchanged in the presence of Spn. Further, Spn stability was moderately increased in the presence of H1N1pdm09, although the degree of stabilization differed between airway surface liquid collected from individual patient cultures. These findings are the first to collect both pathogens from the air and in doing so, they provide insight into the interplay between these pathogens and their hosts.

**IMPORTANCE:**

The impact of microbial communities on transmission fitness and environmental persistence is under-studied. Environmental stability of microbes is crucial to identifying transmission risks and mitigation strategies, such as removal of contaminated aerosols and decontamination of surfaces. Co-infection with *S. pneumoniae* is very common during influenza virus infection, but little work has been done to understand whether *S. pneumoniae* alters stability of influenza virus, or vice versa, in a relevant system. Here, we demonstrate that influenza virus and *S. pneumoniae* are expelled by co-infected hosts. Our stability assays did not reveal any impact of *S. pneumoniae* on influenza virus stability, but did show a trend towards increased stability of *S. pneumoniae* in the presence of influenza viruses. Future work characterizing environmental persistence of viruses and bacteria should include microbially complex solutions to better mimic physiologically relevant conditions.

## OBSERVATION

Environmental stability of respiratory pathogens expelled from an infected host is a key factor impacting transmission (1). Previous work has shown that several factors (e.g., humidity, temperature, and solute concentration) influence microbial stability in droplets (2 - 5). Our understanding of how microbes within the same droplets affect persistence is insufficient, as studies often only examine one microbe at a time. The limited work investigating how bacteria alter viral stability have primarily focused on enteric pathogen stability in feces and found that binding of poliovirus to bacteria increased virus stability (6 - 9). However, these studies did not examine how viruses alter bacterial stability. Futhermore, it remains unclear whether multiple microbes exist within the same aerosols, and if so, whether they influence each other to impact environmental persistence.

Bacterial co-infection is a common occurrence for viral respiratory pathogens: bacterial co-infection rates during influenza virus infection in humans range 4.2–32.7% and cause significant illness in critically sick patients (10 - 12). Studies of influenza virus and *S. pneumoniae* (Spn) secondary infection in animals have shown that influenza virus facilitates transmission of *S. pneumoniae* (13 - 15), while *S. pneumoniae* may decrease viral transmission (14, 15). Another group found that *S. pneumoniae* can increase influenza transmission after antibiotic administration (16). A study on the interaction of nasopharyngeal bacteria with influenza virus observed that influenza virus binds *S. pneumoniae* (17), suggesting that these pathogens may travel in the same aerosols. These observations indicate a complex interplay between these pathogens that requires further investigation to understand how their interactions affect environmental persistence and transmission.

### Co-infected ferrets shed H1N1pdm09 and Spn into expelled aerosols

Co-infections can lead to high titers of virus and bacteria in infected hosts (14, 15, 18), suggesting that multiple microbes could be present within expelled respiratory droplets. To characterize environmental shedding of H1N1pdm09 and Spn, ferrets were first infected with H1N1pdm09 and then infected with Spn 2 d later. Nasal washes were collected, and air sampling was performed for 3 days after co-infection.

**Fig 1:**
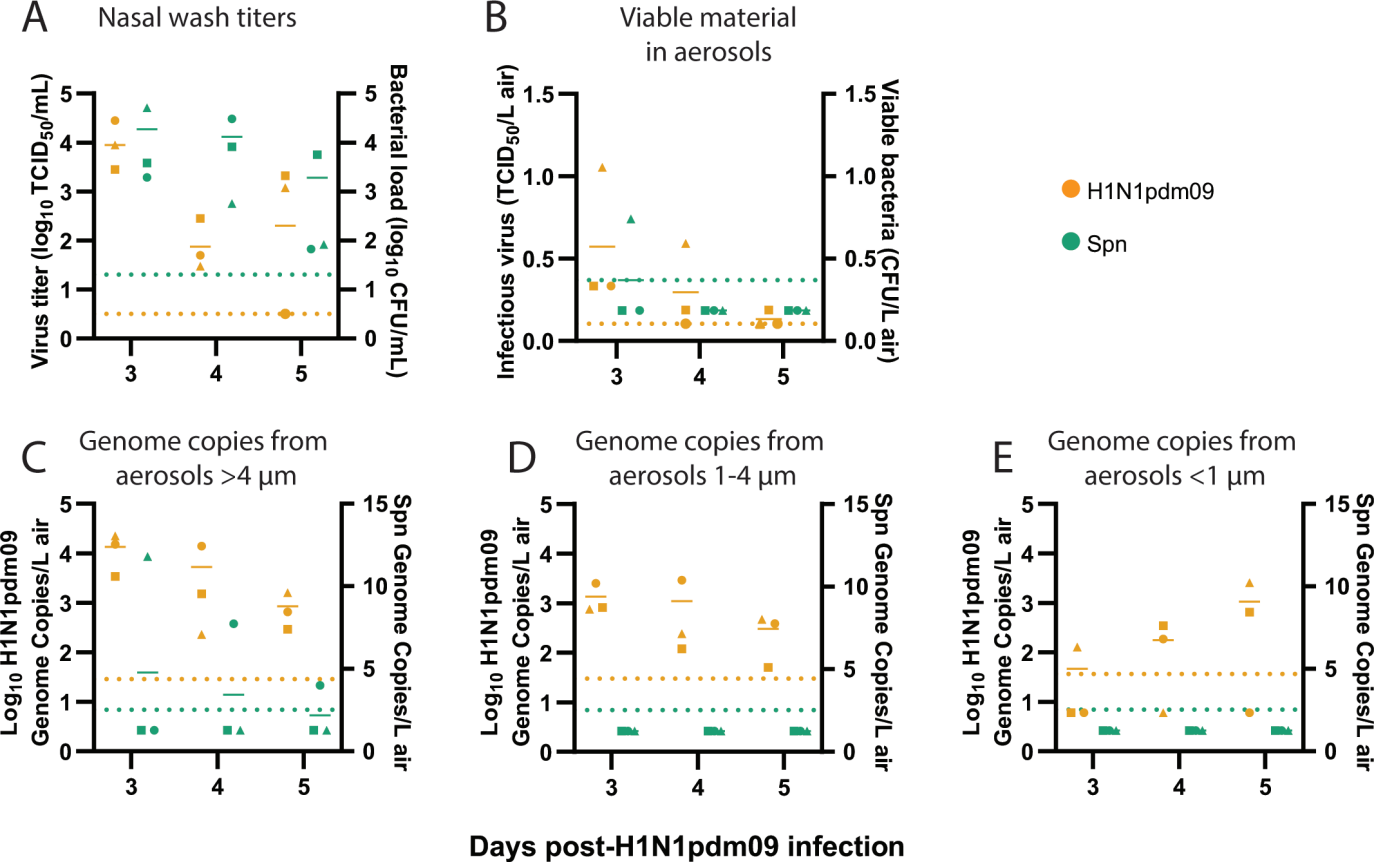
Co-infected ferrets shed H1N1pdm09 and Spn. Ferrets were infected with 10^6^ TCID_50_ of H1N1pdm09 and subsequently infected 2 d later with 10^7^ CFU *S*. *pneumoniae* D39. (**A**) Nasal wash loads of H1N1pdm09 and Spn are shown for the days following initial H1N1pdm09 infection. (**B**) Condensation sampling with a Liquid Spot Sampler was used to collect infectious virus and bacteria shed by co-infected animals. Viral and bacterial loads were measured by TCID_50_ and CFU assays, respectively. (**C–E**) Cyclone-based air samplers were used to fractionate and collect microbial genomic material shed from co-infected ferrets in (**C**) >4 µm droplets, (**D**) 1-4 µm droplets, and (**E**) <1 µm droplets. Quantitative PCR was used to measure genome copies for each microbe. For all graphs, orange symbols represent H1N1pdm09 (*N* = 3) and green symbols represent Spn (*N* = 3), with each animal indicated by a unique shape and the mean indicated by short, solid lines. Dotted lines denote the limit of detection (LOD) for H1N1pdm09 (orange) and Spn (green). Samples without infectious virus were placed at the LOD, and viable bacteria samples below the LOD were placed at 1/2 LOD. Samples without detectable genome copies were placed at 1/2 LOD (see supplemental materials and methods).

Nasal wash titers from co-infected ferrets showed that all three animals shed H1N1pdm09 on days 3 and 4 post-H1N1pdm09 infection, but only two animals shed virus on day 5, while all animals shed Spn throughout the time course (Fig. 1A). We next assessed whether infectious microbes were released from co-infected ferrets by air sampling with a condensation sampler (Fig. S1). Aerosolized infectious H1N1pdm09 was detected from all ferrets on day 3, but from fewer animals on days 4 and 5 (Fig. 1B). Despite measurable levels of Spn in nasal washes, only one animal had viable Spn collected from the condensation sampler (Fig. 1B). This may be under-representing expelled bacteria in the air, as previous work has shown that not all viable bacteria form colonies after aerosolization (19). Cyclone bioaerosol sampling, used to collect microbial genomic material, detected H1N1pdm09 in air samples from all co-infected ferrets for both the >4 µm and 1–4 µm fractions on all days (Fig. 1C and D). The small <1 µm fraction had measurable H1N1pdm09 from one or two of three co-infected ferrets on any day (Fig. 1E). Spn was only detectable in the >4 µm fraction in two animals (Fig. 1C), which is unsurprising given that *S. pneumoniae* ranges from 5 to 10× greater in diameter than influenza and is therefore less likely to be found in smaller aerosols. This result may also under-represent the amount of aerosolized Spn, since sample processing was not optimized for encapsulated bacterial DNA. Our results are the first to detect infectious H1N1pdm09 and viable Spn in expelled aerosols from co-infected animals.

### Environmental stability of H1N1pdm09 is not impacted by the presence of Spn

**Fig 2:**
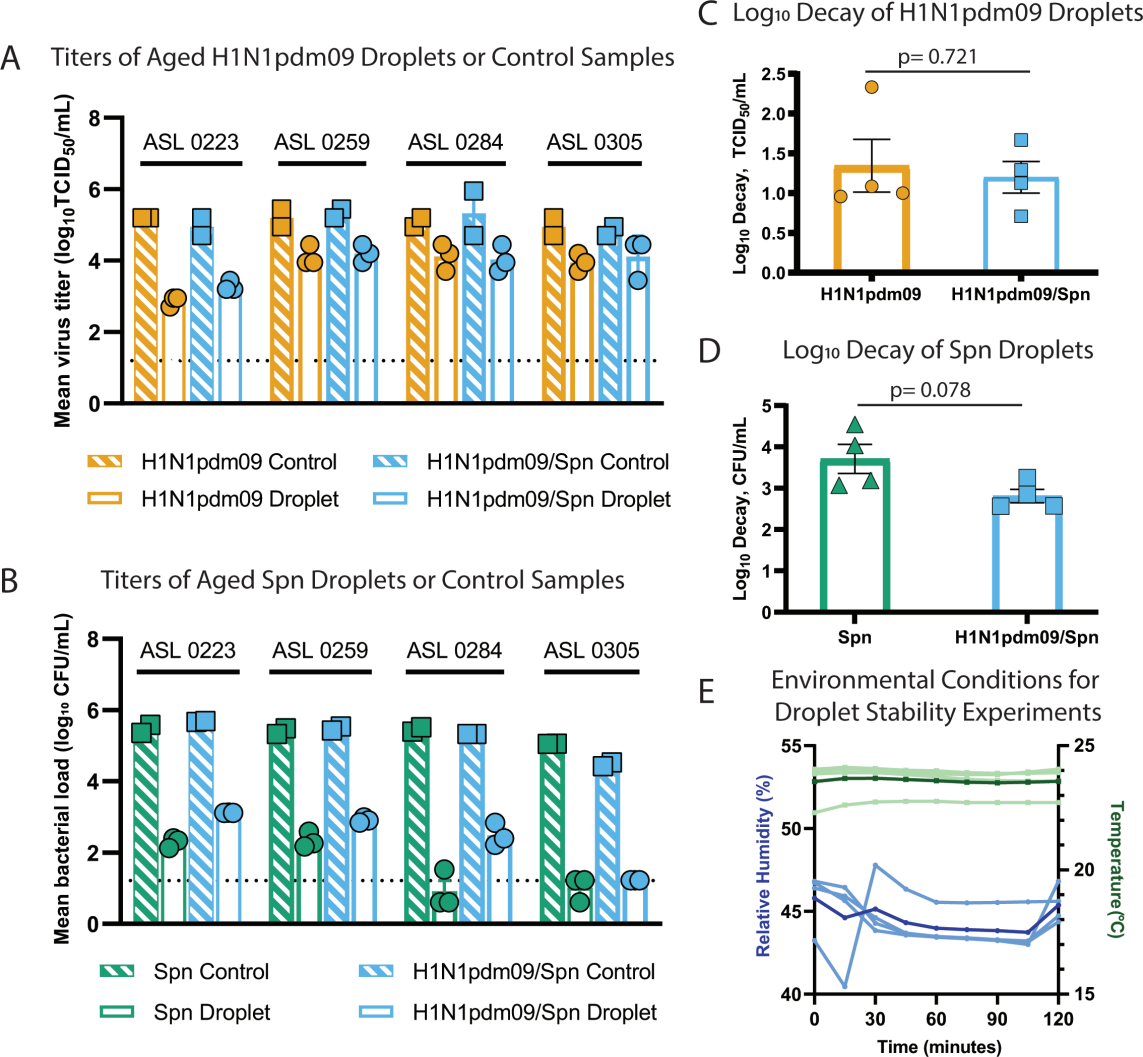
Stability of *S. pneumoniae* and influenza viruses in droplets. (**A–D**) Viral and bacterial loads of H1N1pdm09 and Spn were assessed after exposure of 10 × 1 µL droplets to 43% relative humidity (RH) at room temperature for 2 h. Microbes were suspended in ASL from four different HBE cell donors as indicated in **A** and **B**. Control loads were determined using 10 µL of bulk solutions in closed tubes at room temperature. (**A**) The stability of H1N1pdm09 in droplets containing H1N1pdm09 or H1N1pdm09/Spn measured by TCID_50_ assay, and (**C**) log_10_ decay for each individual ASL culture were determined. (**B**) The stability of Spn in droplets containing Spn or H1N1pdm09/Spn measured by CFU assay, and (**D**) log_10_ decay for each individual ASL culture were determined. Differences were assessed using Welch’s unpaired t-test. (**E**) The RH and temperature were recorded every 15 min during stability experiments. Temperature (light green) and RH (light blue) for each ASL replicate are shown. The average temperature (dark green) and RH (dark blue) for all experiments are also included. Bacterial samples with no detection were placed at 1/2 the LOD.

Given the observation that H1N1pdm09 and Spn are shed from co-infected ferrets, we questioned whether these microbes might influence each other’s environmental stability in respiratory droplets. Spn has been shown to potentially alter influenza A transmission (14, 15), suggesting that Spn might decrease H1N1pdm09 environmental stability. H1N1pdm09, on the other hand, has been shown to increase transmission of Spn (13 - 15), which might indicate enhanced Spn stability with H1N1pdm09. To test this, we measured microbial persistence in droplets containing H1N1pdm09, Spn, or a 1:1 ratio of both pathogens in the presence of airway surface liquid (ASL) collected from four different human bronchial epithelial (HBE) cell donors (Fig. 2). ASL is an important component of the respiratory tract and has been shown to increase stability of influenza viruses in the environment (3). After aging 1 µL droplets of each solution in a humidified chamber for 2 h, infectious H1N1pdm09 or Spn was measured and compared to bulk solution controls (Fig. 2A and B). Experiments were performed at 43% relative humidity (Fig. 2E), as viruses and Gram-positive bacteria are more susceptible to decay at intermediate relative humidity (20). After 2 h, there was no significant difference (*P* = 0.721) in H1N1pdm09 stability with or without Spn (average decay of 1.19 log_10_ TCID_50_/mL versus 1.34 log_10_ TCID_50_/mL, respectively) (Fig. 2C). In contrast, there was a trend of increased stability for Spn in the presence of H1N1pdm09. Improved Spn stability was clearly observed in ASL from one patient culture (284), as Spn alone decayed an average of 3.71 log_10_ CFU/mL and H1N1pdm09/Spn decayed an average of 2.81 log_10_ CFU/mL (*P* = 0.078, Fig. 2D, Table S1). More modest stabilization for Spn was observed in one other culture (223) and no difference was observed in ASL from two patients (259 and 305) (Table S1). Together, these results suggest that H1N1pdm09 infectivity is not impacted by Spn under the environmental conditions tested. There may be a modest impact of Spn stability in the presence of H1N1pdm09, although this may be more sensitive to variations in the ASL (or mucus composition) per individual. Further research should explore the impact of mucus and lung disease states on the relationship between influenza viruses and *S. pneumoniae*.

Co-infection with pathogens can impact the transmissibility to subsequent hosts. Concurrent infections of influenza virus and *S. pneumoniae* result in increased morbidity and a greater risk of bacterial transmission (13, 14). The work here shows that co-infected animals expel both influenza virus and *S. pneumoniae* into air, which can be collected by air sampling. In this study we employed two distinct air sampling methods: a cyclone air sampler (the NIOSH BC251) and a condensation air sampler (Aerosol Devices Liquid Spot). The limitations of these samplers include a short sampling period from 15 min to 1 h, which captures only a snapshot of what is in the air; a lack of detection does not mean that these pathogens are not expelled into the air. In addition to the sampling time, the amount of virus detected in the air will be limited by the flow rate of the specific sampling strategy, the amount of virus expelled by an infected ferret, and the distance of the infected source to the sampling device. These factors contribute to the observed heterogeneity in microbial detection observed in our study and necessitate the need for thoughtful use of specific air sampling devices or incorporation of multiple air sampling devices at varying flow rate and times.

No impact was observed for influenza virus stability in the presence of *S. pneumoniae*, but a trend towards increased *S. pneumoniae* stability in the presence of influenza virus may help explain augmented *S. pneumoniae* transmission in addition to the increased bacterial shedding observed during co-infection (13, 14). Investigation of microbial stability using polymicrobial populations is not widely performed and could help elucidate the complexity of pathogen transmission seen in the human population. In addition, identifying specific host factors underlying microbial stability in the environment could increase our understanding of individual transmission risks and strategies mitigating the spread of pathogens. At this point, the relationship between pathogen detection in air samples, transmission frequency, and virulence remains to be investigated. In the long term, it may be possible to perform air-sampling of patients with identified co-infection to characterize microbes present in respiratory expulsions. Such studies may provide insight into complex transmission events and improve infection prevention measures.

## MATERIALS AND METHODS

Methods can be found in the supplemental material.

## SUPPLEMENTAL MATERIAL

Figure S1 msphere.00039-23-s0001.eps The Liquid Spot condensation sampler was used to collect infectious material from co-infected animals. Co-infected animals were placed in a sampling box for 15 min while sampling was performed. Anti-static tubing connected the sampling box to the inlet of the sampler.

TABLE S1 msphere.00039-23-s0002.pdf Average log decay for H1N1pdm09 or Spn in droplets.

Supplemental materials and methods msphere.00039-23-s0003.docx Supplemental text.

ASM does not own the copyrights to Supplemental Material that may be linked to, or accessed through, an article. The authors have granted ASM a non-exclusive, world-wide license to publish the Supplemental Material files. Please contact the corresponding author directly for reuse.
