# Supplementary material for: Detection of influenza virus and Streptococcus pneumoniae in air sampled from co-infected ferrets and analysis of their influence on pathogen stability
Source: mSphere. 2023 May 31;8(4):e00039-23. doi: 10.1128/msphere.00039-23 (PMC10449498; doi:10.1128/msphere.00039-23)
Supplement: TABLE S1 — Average log decay for H1N1pdm09 or Spn in droplets. [file msphere.00039-23-s0002.pdf]

Supplemental Table 1. Average log decay for H1N1pdm09 or Spn in droplets					
		Log_{10} Decay H1N1pdm09 ± SEM		Log_{10} Decay Spn ± SEM	
ASL Donor	HBE Donor Condition	H1N1pdm09	H1N1pdm09/Spn	Spn	H1N1pdm09/Spn
223	COPD	2.333 ± 0.068*	1.667 ± 0.068*	3.187 ± 0.067*	2.565 ± 0.002*
259	COPD	1.083 ± 0.136	1.125 ± 0.118	3.071 ± 0.101	2.577 ± 0.033
284	IPF	0.958 ± 0.180	1.292 ± 0.180	4.544 ± 0.248*	2.848 ± 0.148*
305	COPD	1.00 ± 0.118	0.708 ± 0.272	4.034 ± 0.167	3.256 ± 0

An asterisk indicates FDR p-value <0.05 when comparing droplets of individual microbes to droplets with both microbes using Welch's unpaired t-tests with Benjamini-Hochberg correction for multiple comparisons.
