# Supplementary material for: Detection of influenza virus and Streptococcus pneumoniae in air sampled from co-infected ferrets and analysis of their influence on pathogen stability
Source: mSphere. 2023 May 31;8(4):e00039-23. doi: 10.1128/msphere.00039-23 (PMC10449498; doi:10.1128/msphere.00039-23)
Supplement: Supplemental materials and methods — Supplemental text. [file msphere.00039-23-s0003.docx]

**Supplemental Materials and Methods**

Virus and Bacteria

A/California/07/2009 (H1N1pdm09) was grown in minimum essential media in Madin-Darby Canine Kidney (MDCK) cells at 37°C for 48 hours and collected by centrifuging supernatant to remove cell debris. Quantification of virus was performed using the 50% tissue culture infectious dose assay (TCID_50_) of 10-fold serial dilution on MDCK cells in 96-well and/or 24-well plates with subsequent assessment for cytopathic effects 4 days after plating. Samples with no detectable virus were placed at 1.2 log_10_(TCID_50_/mL) if titered on 96-well plates or 0.5 log_10_(TCID_50_/mL) if titered on 24-well plates, in accordance with the Spearman Karber method for TCID_50_ determination (21).

*S. pneumoniae* D39 (Spn) was grown in Columbia broth at 37°C. Quantification of bacterial burden was performed by plating 10-fold serial dilutions on blood agar plates and counting colony-forming units after incubation at 37°C overnight. Samples with no detectable bacteria were placed at ½ the LOD to differentiate between samples at the LOD and those with no detection.

Animals

Experiments involving ferrets were performed at the University of Pittsburgh under BSL2 safety conditions (IACUC protocol 19075697). Four to six-month male ferrets were confirmed to be seronegative for influenza infection prior to purchase. Animals were intranasally infected with 10^6^ TCID_50_ of H1N1pdm09 in 500 µL total volume and 10^7^ CFU of Spn in 500 µL. Ferrets were sedated using isoflurane prior to nasal wash collection, performed by collecting the flow-through of PBS passed through the nostrils.

Air Sampling

Infectious virus and bacteria were collected using the Liquid Spot Sampler (Aerosol Devices Inc, Series 110), which uses condensation to collect aerosols into a collection vial. Air was collected from infected animals in a 7 liter chamber connected to the Spot sampler via anti-static tubing for 15 minutes each day at a rate of 1.4L/minute (Supplemental Figure 1). Sampling was performed on days 3, 4, and 5 post-H1N1pdm09 infection (days 1, 2, and 3 post-Spn infection) and prior to nasal wash collection. Condensed aerosols were collected in 700µL 0.5% BSA in PBS. Samples were immediately plated to quantify expelled bacteria and the remaining sample was used for virus titration as described above.

Aerosol sampling of H1N1pdm09/Spn-infected ferrets was performed using cyclone-based air samplers (BC251 developed by NIOSH) on days 3, 4, and 5 post-H1N1pdm09 infection to collect microbial genomic material. Samplers, calibrated to collect 3.5L of air per minute, were placed downwind of infected animals in cages with directional airflow and were run for 1 hour. Samplers fractionated aerosols into three sizes: aerosols >4µm, 1-4µm, and <1µm diameter. After aerosol collection, samplers were washed with isopropanol and allowed to air-dry to avoid contamination.

RNA was isolated using 500µL MagMAX Lysis/Binding Solution Concentrate in each collection tube with thorough vortexing. QIAamp viral RNA mini kit was used to isolate DNA and RNA from lysis solution. Viral and bacterial genome copies were quantified using RT-qPCR with primers against influenza M gene (Forward 5’-AGATGAGTCTTCTAACCGAGGTCG-3’ ; Reverse 5’-GCAAAGACACTTTCCAGTCTCTG-3’ ; Probe 5’-[FAM]TCAGGCCCCCTCAAAGCCGA[3BHQ1] -3’) or *S. pneumoniae* lytA gene (Forward 5’-ACGCAATCTAGCAGATGAAGCA-3’ ; Reverse 5’-TCGTGCGTTTTAATTCCAGCT-3’ ; Probe 5’-[HEX]GCCGAAAACGCTTGATACAGGGAG[BHQ1]-3’). *In vitro* transcribed RNA was used to make a standard curve for influenza virus, and *S. pneumoniae* genomic DNA was serially diluted to generate a standard curve for Spn. Limits of detection were determined by a Ct = 40 or a positive day 0 sample. Samples without amplification in both wells or with Ct greater than the day 0 sample were considered as below the LOD and therefore placed at ½ LOD. qPCR was run using iTaq Universal Probes One-Step kit for influenza (Bio-Rad) and SsoAdvanced Universal Probes Supermix for *S. pneumoniae*. Influenza was amplified for 10 minutes at 50°C, 2 minutes at 95°C, then 40 cycles of 10 seconds at 95°C and 20 seconds at 60°C. *S. pneumoniae* was amplified for 10 minutes at 95°C, and 40 cycles of 15 seconds at 95°C and 1 minute at 60°C.

Stability Experiments

Inside a biosafety cabinet, a saturated salt solution of K_2_CO_3_ was used to condition a glass chamber to 43% relative humidity, and a HOBO UX-100-011 logger was used to record temperature and humidity conditions during each ASL replicate (Figure 2E). Experimental solutions were generated using 10^7.15^ CFU/mL Spn, 10^7.15^ TCID_50_/mL H1N1pdm09, and a 1:5 dilution in PBS of airway surface liquid collected from human bronchial epithelial cells. Ten 1 µL droplets were incubated on polystyrene tissue culture plates in the conditioned chamber for two hours. Controls were 10 µL samples of each microbial solution in closed tubes that were incubated for 2 hours at ambient temperature during the chamber experiments. Log_10_ decay was calculated as previously described and represents the loss in virus or bacterial infectivity (22). Log_10_ decay was determined for each droplet replicate by subtracting the titer of the droplets from the average of the controls for the corresponding ASL. Experiments were performed using technical triplicates for droplets and technical duplicates for controls.

Human lung tissue collected using an approved protocol was used to differentiate human bronchial epithelial cells as previously described (23). Airway surface liquid was collected by washing differentiated cells with 150 µL PBS and collecting the wash (3). All HBE donors were diagnosed with chronic obstructive pulmonary disease (COPD), except for HBE 0284, which came from a patient diagnosed with idiopathic pulmonary fibrosis.

**Data Availability**

The data that supports the findings shown here will be made openly available in FigShare at DOI: 10.6084/m9.figshare.22129055 upon publication. Some of the stability experiments were previously made available on BioRxiv at https://doi.org/10.1101/2020.11.10.376442
